# The hidden malaria: Misidentification of Plasmodium malariae and Plasmodium ovale in low-transmission setting targeted for elimination in Ethiopia

**DOI:** 10.21203/rs.3.rs-9131091/v1

**Published:** 2026-04-09

**Authors:** Hallelujah Getachew, Chloe Wang, Kassahun Habtamu, Assalif Demissew, Teshome Degefa, Solomon K. Birhanie, James W. Kazura, Christopher L. King, Guiyun Yan, Delenasaw Yewhalaw

**Affiliations:** Jimma University; University of California; Menelik II Medical & Health Science College; Ambo University; Jimma University; West Valley Mosquito and Vector Control Agency; Case Western Reserve University; Case Western Reserve University; University of California; Jimma University

**Keywords:** Plasmodium malariae, Plasmodium ovale, species misidentification, qPCR, Ethiopia

## Abstract

**Background:**

A quartan malaria caused by *Plasmodium malariae* (Pm) and a tertian *Plasmodium ovale* (Po) malaria has been largely overlooked in Ethiopia due to milder clinical symptoms, lower prevalence, and the frequent misdiagnosis of these species during routine microscopic. This study estimated the prevalence of Pm and Po infections misdiagnosed as *P. vivax* (Pv) in selected health facilities in southwestern Ethiopia.

**Methods:**

A cross-sectional study was conducted among study participants who were positive for Pv by microscopy or rapid diagnostic test (RDTs) and who were attending health facilities within Arjo-Didessa sugarcane plantations and surrounding areas in Oromia, southwestern Ethiopia, between September 2019 and July 2022. Of the 3590 febrile patients attending health facilities, 323 were Pv positive by microscopy or RDTs. Among these, 245 Pv positive samples were re-evaluated using quantitative polymerase chain reaction (qPCR). Species-specific primers targeting Pv, *P. falciparum* (Pf), Pm and Po were used for species confirmation.

**Results:**

From 245 vivax malaria cases, RDTs was performed to 162 sub-samples, among them 8.6% (14/162) were negative by RDTs. None of the samples tested by RDTs and microscopy detected Pm or Po. Of these 245 tested Pv positive by microscopy was confirmed by PCR, among them 215 (87.7%, 95% CI: 82.98–91.58) were positive for Pv, 9 (3.7%, 95% CI: 1.69–6.86) for Pf, 8 (3.3%, 95% CI: 1.42–6.33) for Pm, 6 (2.4%, 95% CI: 0.90–5.25) were positive for Po and 7 (2.8%, 95% CI: 1.16–5.80) had mixed infections (Pf and Pv).

**Conclusion:**

the findings of this study highlights the need for a strategic shift to integrate Pm and Po into Ethiopia's malaria control and elimination program. Moreover, strengthening the diagnostic capacity by including training microscopists for accurate *Plasmodium* species identification and adopting highly sensitive diagnostic tool for all four *Plasmodium* species in resource-limited settings. This is essential for enhancing malaria control and accelerating progress toward elimination.

## Introduction

Even though, *Plasmodium malariae* (Pm) and *Plasmodium ovale* (Po) *sensu lato* (*s.l*) malaria endemic across tropical and subtropical regions, coexisting with other *Plasmodium* species [1]. The current malaria control and elimination strategy mainly focuses on *Plasmodium falciparum* (Pf) and to a lesser extent on *Plasmodium vivax* (Pv) [2]. *Plasmodium malariae and* Po largely overlooked due to milder clinical symptoms, lower prevalence and the frequent misdiagnosis of these species during routine microscopic examination. In addition, the control of Pm and Po is challenging due to unavailability of Pm or Po species-specific routine diagnostic methods, low-grade parasitemia, and asymptomatic nature of the infection [3–6].

*Plasmodium malariae* clinical manifestation can vary widely, from asymptomatic to severe form of infection. The severe manifestation associated with nephrotic syndromes in young children and adults [7–11] and more long-lasting and chronic illness associated with anaemia and even mortality [12, 13]. A case report of severe manifestation of Po was also documented from central India and Malaysia [14, 15]. In recent years, the incidence of Pm and Po, has increased across the sub-Saharan Africa. This shows the invasion of these parasites could be Duffy antigen independent pathways [16, 17]. Molecular surveys have confirmed co-circulating Pm and Po, suggesting the rising prevalence in some regions where Pf has declined [2, 4, 6, 18, 19]. For instance, an unusually high proportions of Pm clinical cases have been reported in Colombian Amazon Region (43.8%; 294/675) [20] and in Vietnam (46%; 164/356) [21]. This suggests that the transmission patterns of the non-falciparum species do not necessarily follow those of Pf, stressing the need for attention towards Pm and Po malaria [2].

In Ethiopia, the two predominant *Plasmodium* species are Pf and Pv with a general proportion of 60–70% and 30–40%, respectively [22]. *Plasmodium malariae* and Po are considered to be present at a very low prevalence with focal and localized distribution, and often overlooked. The first report on the presence of Pm in Ethiopia was documented in 1938 in southern Ethiopia [23], and then later reported in the late 1960s in western and northwestern areas of the country [24, 25]. On the other hand, the presence of Po was reported from south and northwestern Ethiopia in the late 1960s [24]. More recently molecular studies indicated the presence of both Pm [26] and the two genetically distinct forms of Po in the country [27, 28]. Therefore, the focus of malaria diagnosis, treatment and control strategy need to consider the importance of Pm and Po in the country especially in areas targeted for malaria elimination. Malaria elimination requires the disruption of onward transmission by detecting every infection and proper case management [29]. Accurate species identification and understanding of parasite species distribution are thus important to ensure efficacy of malaria elimination efforts.

In Ethiopia, malaria diagnostic approach varies across levels of the health care system. Health centers and hospitals perform by microscopy, while health post uses rapid diagnostic tests (RDTs) for the diagnosis of Pf and Pv. At the national and regional referral level, molecular test is performed [30]. Although, RDTs require minimal training, no electricity and rapid compared to microscopy [31] RDTs has limitation on the performance for the detection of Pm and Po malaria [32–34] and do not quantify parasite. In resource limited setting microscopy remains the golden standard for malaria diagnosis. An expert microscopist can detect up to 5–10 parasites per μl of blood, while an average microscopist may detect > 100 parasites per μl. [35]. However, microscopic diagnosis has often been challenged by the misidentification of Pm and Po because it requires continuous training to ensure proper diagnosis. On the other hand, malaria RDTs are reported to detect > 100 parasites per μl, whereas molecular tests such as PCR can detect < 5 parasites per μl [35]. It is often not feasible to use the highly accurate and precise technique such as PCR at the primary health care system because it requires a special training, advanced laboratories and resource intensive [17, 36]. Therefore, the aim of this study was to estimate the prevalence of Pm and Po infections misdiagnosed as Pv in selected health facilities in southwestern Ethiopia.

## Methods

### Study area and design

A health facility-based study was conducted targeting febrile patients attending seven selected health facilities around Arjo-Didessa sugarcane irrigated area and the surrounding localities in southwest Ethiopia (8°36′0” N, 36°24′0” E). The study was carried out between September 2019 and July 2022. A detailed description of the study site has been published elsewhere [37]. Malaria transmission in the study area is largely seasonal, low and unstable, with peaks from September to December and minor surge from April to June [38].

### Study participants

Study participants were recruited from the routine health facilities visits; health posts and health center. Self-presenting febrile patients attending Arjo-Didessa Sugar Factory Clinic (n = 1690), Command 2 Health Post (n = 260), Command 5 Health Post (n = 200), Abote Didessa Health Post (n = 250), Hunde Gudina Health Post (n = 430), Kerka Health Post (n = 400), and Sefera Tabiya Health Post (n = 360) were included. Patients above the age of 6 months and attending the selected health facilities from September 2019 to July 2022 were eligible for the study. Any patient reported as being positive for Pv using microscopy or RDTs were asked for voluntary participation in the study. During enrolment, trained data collectors administered a structured questionnaire to the consenting Pv mono-infected participants to obtain socio-demographic information and data on malaria control measures. Then capillary blood samples were collected for microscopy and PCR analysis.

### Sampling

A total of 245 capillary blood samples were collected on filter papers from microscopy or RDTs confirmed vivax malaria outpatients for molecular diagnosis during the study period. The collected capillary blood sample on filter papers were air-dried, placed in separated plastic bags containing silica gel, and stored at −80°C for further analysis.

### Rapid diagnostic test (RDTs)

Capillary blood sample was collected by finger prick and 5μl blood was added into the sample well of multi-species CareStart^™^ Malaria Pf/Pv (HRP2/pLDH) Ag Combo RDT test kits (Access Bio Ethiopia, INC.) that detects PfHRP2 and PvLDH. Then two drops (60μl) of buffer were added into the buffer well. Finally, the test result was read in 20 min. Interpretation of the results were done according to the manufacturer instructions.

### Microscopic examination

Thick and thin blood films were prepared and stained with 10% Giemsa solution and a medical laboratory technologist examined under light microscope at Arjo-Didessa Sugar Factory Clinic. The normal protocol for microscopic diagnosis at the health facilities was followed. Parasite density was not evaluated because the routine work of health facilities in rural areas of Ethiopia involves checking for the presence or absence of parasites but does not extend to calculating parasite densities. A second highly trained medical laboratory technologist from Jimma University rechecked all positive slides.

### DNA extraction

For the DNA extraction procedure, modified Chelex-100 resin was utilized [39]. Using a puncher, one dot of the dried blood spot from filter paper was cut into piece that was roughly 3–5 mm in size. After each sample, the puncher was cleaned by punching paper ten times. After transferring the blotted filter paper to a 1.5 ml Eppendorf tube, 50 μl of 10% saponin and 950 μl of phosphate buffered saline (PBS) were added to lyse the red blood cells. The sample was mixed and then incubated for four hours or overnight at 4°C. After centrifuging the mixture for 10 minutes at room temperature at 14,000 rpm, the supernatant was removed. After adding 1 ml of PBS and centrifuging at 14,000 rpm for roughly 5 min, any remaining saponin was discarded. By centrifuging for roughly 15 seconds, the leftover PBS was discarded. The filter paper was allowed to dry at room temperature for 15 min. To extract the parasite DNA, 150 μl of 20% Chelex suspension and 100 μl of distilled water was added. The mixture was then incubated at 95°C in a water bath for 10 minutes, vortexing it every two minutes. After centrifuging the mixture for one minute at 14,000 rpm, 200 μl of the supernatant (DNA) was transferred to a 0.5 ml tube and kept at −20°C for qPCR analysis.

### Plasmodium species identification using multiplex qPCR

Identification of *Plasmodium* species using multiplex qPCR. Each DNA sample's was amplified using primers based on 18S rRNA genes. Two round of amplification was carried out. Round one was used to identify Pv and Po. Second round was to Pv and Pm. In round one a total of 12 μl qPCR reaction mixture containing 2.1 μl genomic DNA, 6 μl FastAdvanced MM (2X), 0.4 μl forward and reverse primers for Pf, Pv, and Po, each (Var-F, Var-R, Pv-1, Pv-2, Po-1, and Po-2), and 0.5 μl of each probe (Pf_varAST_Probe, Pv-Probe, and Po-probe) for Pf, Pv, and Po, respectively [40, 41]. In second round, a total of 12 μl qPCR reaction mixture containing 2.1 μl genomic DNA, 6 μl FastAdvanced MM (2X), 0.4 μl forward and reverse primers for Pf, Pv and Pm, each (Pf-1, Pf-2, Pv-1, Pv-2, Pm-1, and Pm-2) then 0.5 μl of each probes (Pf18S_Probe, Pv-Probe, and Pm-probe) for Pf, Pv and Pm. Quant Studio 5, Applied Biosystems Real-Time PCR, was used for this analysis with a reaction time an initial hold stage at 50°C for 2 min and 95°C for 2 min, followed by 45 cycles of qPCR amplification stage at 95°C for 3 s and 60°C for 30 s. for each round 1 and round 2 amplification process. Sequences of primers are shown in Supplementary Table S1.

### Data processing and analysis

Data were checked for completeness and consistency before analysis; then all the data from study participants were imported into Excel. The statistical analysis was performed by STATA (version 17) and Graph-Pad Prism (version 9.5.1). Descriptive statistics were used to assess the distribution of the sociodemographic characteristics and independent variables as well as frequencies and proportion of malaria-positive samples with 95% confidence intervals (CIs). In addition, chi-square (χ^2^) test (replaced by Fisher’s exact test if necessary) were used.

## Results

Of the 3,590 febrile patients attending health facilities, 323 were Pv positive by microscopy or RDTs out of which 245 Pv case were re-evaluated by PCR. Among the 245 cases enrolled in the study, the median age of the study participants was 20 (interquartile range (IQR) 13.5–28). The majority (64.9%) were adult > 15 years of age and 64.1% were male (Table 1).

All 245 samples were confirmed to be *Plasmodium*-positive by qPCR. Among these 245 qPCR-confirmed cases, 215 (87.7%) were positive for Pv, 9 (3.7%) for Pf, 8 (3.3%) for Pm, 6 (2.5%) were positive for Po, and 7 (2.8%) had mixed infections (Pf and Pv) (Figure). All Pm and Po infections were detected only by PCR while undetected in microscopic examination or RDTs. When the samples were stratified by patient age, the majority of Pm and Po infections were in adult > 15 years of age (11/159, 6.9% infection rate). However, males (13/157, 8.3%) and those worked outdoors (12/136, 8.8%) were affected. In addition, infection was higher (8/75, 10.7%) in dry season (Table 2).

### Comparison among diagnostic methods

From the 245 vivax malaria cases, RDTs was performed for 162 sub-samples, among them 8.6% (14/162) were negative by RDTs.(Supplementary Table S2). From the RDTs performed cases qPCR were confirmed and 144 (88.8%, 95% CI: 83.01–93.28) were positive for Pv, 6 (3.7%, 95% CI: 1.37–7.89) Pf, 5 (3.1%, 95% CI: 1.01–7.06) Pm, 2 (1.2%, 95% CI: 0.15–4.39) Po and 5 (3.1%, 95% CI: 1.01–7.06) mixed infection (Pf and Pv) (Table 3). Moreover, all 245 microscopically examined cases were confirmed by qPCR. Of these 245 PCR-confirmed cases, 215 (87.7%, 95% CI: 82.98–91.58) were positive for Pv, 9 (3.7%, 95% CI: 1.69–6.86) Pf, 8 (3.3%, 95% CI: 1.42–6.33) Pm, 6 (2.4%, 95% CI: 0.90–5.25) Po, and 7 (2.8%, 95% CI: 1.16–5.80) mixed infection (Pf and Pv) (Table 3).

## Discussion

Malaria elimination entails interruption of onward transmission by detecting every infection and undertaking proper case management [29]. Accurate parasite species identification is the cornerstone of proper diagnosis, treatment, case management, and malaria intervention. This is also important in understanding the epidemiology as well reduces the occurrence of drug resistance [26, 42]. In this study, among 245 PCR-confirmed *Plasmodium* positive cases, 3.3% Pm and 2.4% Po were detected. The present study reported the occurrence Pm for the first time in southwestern Ethiopia, while Po was previously reported by the same study team in 2019 community based study as a submicroscopic infection [38]. This study also showed the high prevalence of these two *Plasmodium* species in adult males and individuals who were engaged in outdoor activities. In addition, these rare *Plasmodium* species occurred during the dry season of the year unlike the common *Plasmodium* species that occur during the end of the rainy season.

The misidentification of Pm and Po by microscopy and RDTs could lead to underestimate the true burden of these parasites. Historically Pm and Po infections were presumed to be less than 1% in Ethiopia [30]. In this study, all Pm and Po infections were misidentified as Pv malaria by microscopy. In Ethiopia, microscopy is considered as the gold standard for malaria diagnosis. However, Pm and Po are commonly misdiagnosed by microscopy since microscopists are not often trained with rare *Plasmodium* species [30]. Different studies in Ethiopia also demonstrated misidentification of Pm and Po in the northwest [27], south [26] and southeastern Ethiopia [28]. This indicates that there is a need for new diagnostic tools with high specificity and accuracy to drive malaria elimination in low transmission settings like Ethiopia.

The finding of this study also revealed that higher prevalence of Pm and Po was recorded in adult male and outdoor workers. The result of this study was supported by a study from Papua Indonesia where Pm infection was higher among adults above 15 years of age [13]. However, studies in sub-Saharan Africa indicated that most of Pm and Po infection were documented in young children less than 15 years of age [3, 5, 18, 43–46]. In line with the present study, studies in Côte d’Ivoire and Mali reported dry season transmission of these malaria parasites [45, 46]. However, another study in Kenya showed no seasonal difference in the occurrence of both Pm and Po [47] while a study in Tanzania demonstrated transmission at the beginning of wet season [48]. More importantly, Pm is known to cause chronic, low-density infections that can persist for years, acting as a durable reservoir that can bridge dry seasons when *Anopheles* mosquito density is low. Po possesses hypnozoites, causing clinical relapses months or years after the infective bite, without new mosquito exposure. These biological features mean that transmission can be sustained sporadically and silently.

In this study, microscopic diagnosis misclassified 13 malaria cases (5.3%) as Pv mono-infections, while qPCR identified them as Pf mono-infections (7 cases) and Pf/Pv mixed infections (6 cases). Such misclassification were also observed in the RDTs in this study. Consequently, those patients were inappropriately treated with chloroquine instead of the recommended artemisinin-based combination therapy (ACT) and single dose primaquine as per national guidelines. Such misclassification could potentially contribute to a reduced treatment efficacy and increased risk of severe malaria consequences, including mortality. The omission of a buffer in the preparation of Giemsa stain working solution across all laboratories in the study area could be a possible justification for misclassification of the parasites. Optimal staining of Giemsa can be attain by maintaining the pH of Giemsa, as it ensures clear visualization of parasite species-specific morphological features, such as Schüffner’s dots in Pv and Maurer’s clefts in Pf, which are essential for correct species identification. Similar findings linking inadequate staining to reduced microscopic sensitivity and species misidentification have been reported elsewhere in Ethiopia [26, 28]. A key to an effective case management is an accurate species identification and proper treatment. Therefore, misidentification directly undermines treatment efficacy and can sustain transmission.

This study was not without limitation. The study used only Pv samples however, negative samples, Pf positive and mixed infection (Pf and Pv) were not included. If these samples were included the prevalence might differ. The genetic diversity of Po was not done to identify *P. ovale wallikeri* and *P. ovale curtisi.*

In conclusion, this study highlight the needs for a strategic shift to integrate Pm and Po species into Ethiopia's elimination program. First, strengthen the diagnostic capacity, this includes a revised microscopy training modules by incorporating Pm and Po proper diagnosis, and the strategic deployment of a highly accurate diagnostic tool for all *Plasmodium* species that can be applied at the primary health care system. Secondly, all other *Plasmodium* species beyond the Pf and Pv must be identified and documented explicitly in the surveillance systems. Moreover, targeted screening of an outdoor labourers residing in irrigation development areas and sustained malaria surveillance both in high transmission season (wet season) and low transmission (dry season). Finally, in order to achieve malaria elimination, treatment must ensure that any Pm or Po infection, once confirmed, should receive appropriate radical treatment in order to achieve sustainable malaria elimination in Ethiopia.

## Supplementary Material

This is a list of supplementary files associated with this preprint. Click to download.

• Supplementary.docx

## Figures and Tables

**Figure 1:**
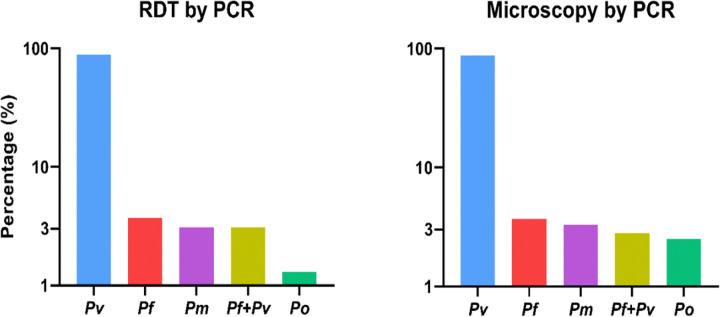
Percentage (logarithmic vertical scales) of Pv, Pf, Pm, mixed infection Pf+Pv and Po cases by microscopy (245 samples) and RDTs (162 samples) confirmed by PCR in Arjo-Didessa sugar development area, southwest Ethiopia

**Table 1:** Sociodemographic characteristic of the study participants (n = 245)

Variable	N	%
**Age (in years)**		
Median (IQR)	20(13.5–28)	
Mean (± SD)	21.6(± 12.7)	
≤ 15	86	35.1
> 15	159	64.9
**Sex**		
Female	88	35.9
Male	157	64.0
**Education**		
≥ Secondary	77	31.4
Primary	91	37.2
No education	77	31.4
**Work place**		
Indoor	109	44.5
Outdoor	136	55.5
**Duration of stay in the area**		
≤ 3year	140	57.1
> 3 years	105	42.9
**Season**		
Dry	75	30.6
Wet	170	69.4

**Table 2:** Sex and age specific *Plasmodium* prevalence among 245 study participants in Arjo-Didessa sugar development area, southwest Ethiopia, 2019–2021

Variable	Total enrolled	*Pv*, n(%)	*Pf*, n(%)	*Pm*, n(%)	*Pf + Pv*, n(%)	*Po*, n(%)
**Sex**						
Female	88	79 (89.8)	3(3.4)	1(1.1)	5(5.7)	0
Male	157	136 (86.6)	6(3.8)	7(4.5)	2(1.3)	6(3.8)
**Age group** (in year)						
≤ 15	86	78(90.7)	2(2.3)	1(1.2)	3(3.5)	2(2.3)
> 15	159	137(86.2)	7(4.4)	7(4.4)	4(2.5)	4(2.5)
**Work place**						
Indoor	109	103(94.5)	2(1.8)	0	2(1.8)	2(1.8)
Outdoor	136	112(82.4)	7(5.2)	8(5.9)	5(3.7)	4(2.9)
**Season**						
Dry	75	59 (78.7)	6(8.0)	4(5.3)	2(2.7)	4(5.3)
Wet	170	156(91.8)	3(1.8)	4(2.4)	5(2.9)	2(1.2)

**Table 3:** Comparison of the performance of RDTs, microscopy and qPCR for malaria diagnosis in Arjo-Didessa sugar development area, southwest Ethiopia, 2019–2021

Diagnostic		qPCR					
methods		Pv, n(%)	Pf, n(%)	Pm, n(%)	Pf + Pv, n(%)	Po, n(%)	Total
**RDT**	**Negative**	14 (100)	0	0	0	0	14
	**Pv**	130 (87.8)	6 (4.1)	5 (3.4)	5 (3.4)	2 (1.4)	148
	**Total**	144 (88.9)	6 (3.7)	5 (3.1)	5 (3.1)	2 (1.2)	162
**Microscopy**	**Pv**	215 (88.8)	7 (2.9)	8 (3.3)	6 (2.5)	6 (2.5)	242
	**Pf**	0	1 (100)	0	0	0	1
	**Pf + Pv**	0	1 (50)	0	1 (50)	0	2
	**Total**	215 (87.7)	9 (3.7)	8 (3.3)	7 (2.9)	6 (2.4)	245

## Data Availability

Deidentified participant datasets used for the current study will be available immediately after publication from the corresponding author on reasonable request.
